# The Protective Effect of Abortion on Preeclampsia: An Analysis of Current Research

**DOI:** 10.7759/cureus.54131

**Published:** 2024-02-13

**Authors:** Sarah J Norman, Gena Fontus, Chancée Forestier, Tasneem Hiba, Stephanie Colon Pagan, Michael Osondu, Volha Shylovich

**Affiliations:** 1 Medicine, American University of the Caribbean School of Medicine, Cupecoy, SXM; 2 Medical School, Ascension Providence Hospital, Southfield, USA; 3 Obstetrics and Gynecology, BronxCare Health System, New York, USA

**Keywords:** preeclampsia, eclampsia, maternal immune tolerance, reproductive immunology, induced abortion, spontaneous abortion, preeclampsia-eclampsia

## Abstract

A review of the current literature on preeclampsia (PE) confirms that this pregnancy complication remains a common cause of maternal mortality. Within the last several decades, obstetric and gynecological researchers worldwide have indicated an association between prior abortions and the development of PE. Different studies have debated whether abortion is a protective or risk factor for PE. However, the most current literature demonstrates a stronger likelihood that a positive history of abortions will offer a protective effect against PE. This association has been supported by advancements in the reproductive immunology literature, which states complex fetal and paternal pathological mechanisms help to build maternal immunological tolerance, thus protecting expectant mothers from pregnancy complications. This literature review will compare studies supporting prior abortions offering a protective effect against PE with those stating prior abortions are a risk factor for the development of PE. Additionally, this critical review will discuss the advancements and current understanding of reproductive immunology and how it pertains to this association between positive abortion history and PE.

## Introduction and background

An overview: Preeclampsia and abortion

Preeclampsia (PE) is a progressive and multifactorial disorder that is considered one of the major complications of pregnancy and is a common cause of maternal mortality. Patients at greater than 20 weeks of gestation are given the diagnosis of PE after a new onset of hypertension is detected with two distinctive blood pressure readings equal to or above 140/90 mmHg that are at least four hours apart [1]. Other criteria include 300 mg of proteinuria or new onset end-organ damage. Proteinuria is defined as 300 mg or more on a 24-hour urine collection, or >1+ on urine dipstick testing. Estimations of proteinuria can also be made with a random urine protein-to-creatinine ratio of >0.3, or an albumin-to-creatinine ratio >9.8 mg/g. Signs and symptoms of severe PE can include hypertension (greater than 160 mmHg systolic or diastolic greater than 110 mmHg), severe refractory headache, visual abnormalities, upper abdominal pain, altered mental status, and difficulty breathing. The combination of severe headache and hypertension can induce a hemorrhagic stroke, a feared complication of PE, which can further lead to serious disability or death. When a seizure occurs in a PE patient, the diagnosis progresses from PE to eclampsia [2].

There are many potential etiologies of PE, and these pathological mechanisms include uteroplacental ischemia, atherosclerosis, breakdown of maternal-fetal immune tolerance (usually seen in primipaternity and egg donation), and syncytiotrophoblast stress [3]. During the first trimester, several critical events occur such as the invasion of trophoblasts and the remodeling of the spiral arteries. Any defects in cytotrophoblast differentiation can greatly reduce the number of trophoblasts available to remodel the spiral arteries [4]. This results in a sequence of defective processes, including insufficient conversion of spiral arteries, inadequate invasion into the uterine wall, and defective endothelial cell apoptosis. Each of these conditions impacts the flow of blood to the placenta. For example, the spiral arteries in a pregnant patient with PE will be half the diameter than those of a patient without PE. This further impairs the blood flow to the placenta further disturbing the oxygen supply. This inadequate perfusion can result in placental hypoxic injury, which generates hypoxia-inducible factor-1 (HIF-1) [4]. This factor degrades glial cell missing-1 (GCM1), thereby increasing syncytial debris shed. The overall increase in debris volume leads to necrosis rather than the usual apoptosis seen in a normal pregnancy. The necrosis induces a surge of proinflammatory cytokines that leads to the many signs and symptoms of PE [4].

After the diagnosis of PE, the main goal is to keep the patient pregnant as long as possible while monitoring and maintaining safe blood pressure using non-teratogenic antihypertensive medications, such as labetalol [5]. Refractory severe blood pressure readings equal to or greater than 160 mmHg systolic or 110 mmHg diastolic are considered a medical emergency and would warrant immediate action. Delivery of the fetus and placenta is the only intervention that leads to disease resolution. During labor, these patients require continuous blood pressure monitoring due to their high risk of placental insufficiency and fetal hypoxic stress. Antihypertensive medication should be continued and prescribed as needed [5]. Postpartum, the patient must be monitored for at least 24 hours for any signs of deterioration or worsening disease. Patient blood pressure monitoring should be continued for one week due to the high percentage of postpartum PE. Postpartum PE is diagnosed with a new onset of hypertension 48 hours to six weeks after delivery; these patients commonly present to the emergency department with neurologic symptoms seven to 10 days post-delivery and are at high risk for developing eclampsia [1].

According to a systematic review by Fingar et al., almost 5% of pregnancies worldwide were significantly affected by PE [6]. In 2014, the incidence of PE in the United States was 4.7% and it was the most common hypertension-related diagnosis in pregnant women. Of those with PE, 37.2% of the deliveries had severe PE [6]. Since then, the incidence of PE has steadily increased due to various population-related increases in risk factors. This makes the topic more concerning and requires an increased amount of research to analyze the causes of these pregnancy complications as well as proper management. The risk factors for PE include a history of PE, pregestational diabetes, chronic hypertension, obesity, chronic kidney disease, and multifetal pregnancy. It is estimated that one-third of the patients diagnosed with PE are nulliparous [7], and two-thirds have had previous pregnancies. Research has begun to investigate the association specifically between prior abortions and developing PE in subsequent pregnancies.

Spontaneous abortion describes pregnancy loss that occurs before 20 gestation weeks. Etiologies of abortions tend to be complicated; however, first-trimester spontaneous abortions have been linked to genetic abnormalities of the fetus, maternal endocrine disorders, including diabetes and thyroid pathologies, immunological disorders, and maternal infections [8]. Spontaneous abortions are further classified as threatened, incomplete, complete, or missed miscarriages. When fetal tissue has not been fully evacuated from the uterus, such as in the case of incomplete or missed abortions, medical or procedural treatment is recommended to avoid infections and other complications. Medical management for abortions includes misoprostol to dilate the cervix, while procedural intervention includes dilation and curettage. Dilation and evacuation are mostly used after 13 weeks gestation during the second trimester and entail a similar procedure as the dilation and curettage.

According to the CDC 2020 data, there were a total of 615,911 abortions among 49 reporting areas (47 states, the District of Columbia, and New York City, excluding California, Maryland, and New Hampshire). During this year, the abortion rate was 11.2 abortions per 1,000 women aged 15-44 years with a ratio of 198 abortions per 1,000 live births. In the years 2019-2020, the incidence decreased by 2% while the abortion ratio increased by 2%, as shown in Table 1.

**Table 1:** Total number of abortions, rate, and ratio in reported areas according to the CDC from years 2015 to 2020. Data are as per the CDC from 49 reporting areas (47 states, the District of Columbia, and New York City, excluding California, Maryland, and New Hampshire) [9].

Year	Total number of abortions	Abortion rate	Abortion ratio per 1,000 births
2015	638,169	11.8	188
2016	623,471	11.6	186
2017	612,719	11.2	185
2018	619,591	11.3	189
2019	629,898	11.4	195
2020	615,911	11.2	198

This decline began in 2011 with an overall decrease in abortions by 15%, a rate decrease of 18%, and a ratio decrease of 9%. There was a minor increase during the 2018-2019 years before a continued decline in 2020 [9]. In 2020, more than half of the abortions (57.2%) reported were from women aged 20-24 (27.945) and 25-29 (29.3%) years, corresponding to the data in previous years (Table 2).

**Table 2:** Percentage of abortions based on maternal age. Percentage obtained by women in a given age group per 1,000 women in the same age group [9].

Age	Percentage of abortions
20-24 years old	27.90%
25-29 years old	29.30%
30-34 years old	19.90%
Other	23.50%

Nearly all abortions in 2020 in the USA per the CDC took place early in gestation: 93.1% of abortions were performed at ≤13 weeks of gestation; with 5.8% performed at 14-20 weeks of gestation, and only 0.9% were performed at ≥21 weeks of gestation [9]. In regards to methods of abortion, 46 areas reported that 51.0% of all abortions were early medical abortions at less than nine weeks, with 2.4% being medical abortions at more than nine weeks. Additionally, 40% were surgical abortions at less than 13 weeks, with 6.7% of surgical abortions at more than 13 weeks (Table 3) [9].

**Table 3:** Methods of abortions and associated gestational age. Data from 40 reporting areas, excluding California, Connecticut, Louisiana, Maryland, District of Columbia, Massachusetts, New Hampshire, New York, Pennsylvania, Illinois, Tennessee, and Wisconsin [9], per the CDC from 2020.

	Less than 9 weeks	More than 9 weeks	Less than 13 weeks	More than 13 weeks
Medical abortions	278,947 (51.0%)	12,943 (2.4%)	—	—
Surgical abortions	—	—	218,734 (40.0%)	36,531 (6.7%)

Abortion is a frequently debated topic in association with PE. Numerous studies within the past several decades have been published characterizing a positive abortion history as a risk factor for PE. However, most current research analyses establish positive abortion history as a protective factor instead. The objective of this literature review is to compare the studies by investigating the association between PE and prior abortions whilst exploring the related reproductive immunology literature and emphasizing the need for further research on the topic.

## Review

Abortions: Risk factor or protective factor for preeclampsia?

PE is a serious and potentially life-threatening pregnancy complication and the leading cause of maternal and perinatal morbidity and mortality worldwide [10]. The association between PE and abortions has been a topic of research and discussion over the last several decades. While some studies have found a history of PE to be a protective factor for the development of PE in future pregnancies, other studies have found that a history of abortions increases the risk of PE in future pregnancies. Additionally, some researchers have found no significant association between abortion and PE.

This systematic review of the literature was completed by researching freely accessible articles online using the following databases: UpToDate, PubMed, Science Direct, Biomed Central, Elsevier, and National Institutes of Health. The inclusion criteria included global studies in reference to prior abortions and PE from 1990 to the present day and further included both primary studies and review articles. Studies before 1990 and studies in a non-English language were excluded. Next, the selection of articles was conducted in two different stages. Firstly, the titles and abstracts of all resources were based on the inclusion criteria and specific keywords such as "preeclampsia" and "abortion." The second stage included screening and reading the selected articles to ascertain if the content was relevant to our main investigation, which was to analyze the association between prior abortions and the risk of PE, and to also discuss prior abortions as a potential protective factor. Furthermore, this literature review has compared studies that met these criteria by acknowledging methods, limitations, and other factors that helped to establish the connection between prior abortions and PE. In addition to this, other risk factors were discussed such as hypertension, chronic kidney disease (CKD), and changes in hemoglobin to better understand the association of these factors in relation to PE.

Prior abortions as a protective factor for preeclampsia

Various pathological mechanisms and risk factors are intricately linked to the development of PE. For instance, prior abortions seem to protect women from developing PE in subsequent pregnancies due to contact between fetal and maternal tissue, which directly contributes to immunological tolerance. Therefore, complications with this interface are one of the possible causes of pregnancy complications such as PE, thus supporting the rationale within current global research that there is an association between prior abortions being a protective factor against PE. This literature review will begin by analyzing crucial studies that were completed in Sudan, Norway, Finland, Canada, and the United States that support prior abortions as a protective factor for PE.

A case-control study conducted in Sudan specifically looked at the relationship between spontaneous abortion and PE among pregnant women [11]. The study consisted of 360 individuals divided using a 1:1 ratio, with the control consisting of healthy pregnant women and the experimental group of pregnant women with clinically diagnosed PE. A study-specific questionnaire was used to assess the women’s obstetrics history and sociodemographic characteristics, and upon further analysis, the findings concluded that a history of spontaneous abortion reduced the risk of PE by 59% [11]. Although the study concluded that spontaneous abortion is a protective factor, one of the important limitations of the study is that it did not control for communicable diseases, such as herpes and toxoplasma, which have been shown to be associated with PE in previous literature. This particular study also admits that there may be some recall bias among participants as most of the pertinent data collected were from the questionnaire [11]. Despite the limitations and smaller sample sizes, similar findings are seen in other studies.

The Norwegian Mother and Child Cohort Study consisted of 20,846 nulliparous women in whom researchers Trogstad et al. sought to estimate the risk of PE for women with a positive history of abortion [12]. Participants also answered a questionnaire at 15 weeks’ gestation that assessed the outcomes of all prior pregnancies, education, health, and habits. For this population, two or more previous induced abortions reduced the risk of PE significantly (OR: 0.36, 95% CI: 0.18-0.73). The study stated that the proposed reasoning for this association may be due to induced maternal immunological changes that interrupt placental physiology and growth as well as the importance of paternal genetic contributions [12].

A 2015 case-control study in Finland investigated 12,560 cases of PE with 50,600 matched controls and found that induced abortion reduced the risk of PE for nulliparous women, but the association was small [13]. This risk reduction was greater in women with induced abortions at 12 weeks or later. Interestingly, the study adjusted for the length of time since the last abortion but did not adjust for the low risk of PE in women with a history of two or more induced abortions. This study also stated that it was unclear if this association is applicable to both surgical and medical-induced abortions [13]. Furthermore, Parker et al. controlled for changes in paternity and found that the risk of PE was reduced in same-paternity pregnancies compared to new paternity pregnancies, which currently supports other studies and current data in the reproductive pathology literature [13].

A study completed by Xiong et al. in Alberta, Canada sought to examine the effects of previous abortions and the incidence of PE in subsequent pregnancies by identifying 140,773 pregnancies between 1993 and 1999 and evaluating the previous medical history and pregnancy outcomes of these women [14]. This study used logistic regression analysis to determine odd ratios within the data of these identified pregnant women. It was found that a single previous abortion was related to a reduction in the development of PE in a subsequent pregnancy (adjusted odds ratio (AOR): 0.84; 95% CI: 0.72-0.97; p < 0.05) [14]. However, neither two nor greater than three prior abortions illustrated any statistical significance in relation to the development of PE. The researchers further declared that the data from the study supported the widely accepted association that the incidence of PE occurs less in multiparous women compared to primigravida women [14]. While the analysis concludes that a single prior abortion is protective for PE, the researchers reiterated that further research is needed to establish an indisputable relationship between these two factors as well as establish the differences between spontaneous and induced abortions in this context.

Saftlas et al. utilized a randomized placebo-controlled clinical trial to examine 11,959 pregnant women in various regions of the United States to determine if calcium supplementation could reduce the risk of the development of PE [15]. This study concluded that women with previous abortions who conceived with a new partner have the same risk as nulliparous women without a history of prior abortions (AOR: 1.03, 95% CI: 0.72, 1.47) [15]. Upon further analysis of the data, the researchers claimed that nulliparous women with previous abortions reduced the risk of PE by 50% if the next pregnancy was with the same partner. However, if there is a changed paternity, the risk of PE is the same as in nulliparous women. This finding suggests that paternal factors are intricately related to the immune tolerance and reproductive mechanisms needed to promote a normal pregnancy. Moreover, men who have a history of fathering a preeclamptic pregnancy have an increased risk of imposing another preeclamptic pregnancy on a new female partner [15]. These findings further establish the need for literature pertaining to the sperm microbiome, the importance of paternal antigens and genetics, and how the fetal and paternal factors are intricately related to one another and placental function.

A cohort study completed by Li & Wi in California, USA specifically focused on the change in paternity in pregnant women with and without PE in their first pregnancy [16]. The study used birth certificate data to identify 140,147 women with two consecutive pregnancies between 1989 and 1991 [16]. Paternity was determined using the surname on the birth certificate and birth certificates without a paternal surname were excluded. The study adjusted for other factors such as maternal age, parity, miscarriage, race/ethnicity, prenatal smoking, and infant sex. The study found that women without PE in the first pregnancy were found to have a 30% increased risk of PE in the second birth if they changed partners. The effects of paternity got more interesting when this study showed that women who had PE during their first pregnancy had a 30% decreased risk of PE if they changed partners [16]. This study suggested that paternal genetics and immunological factors may play a role in the development of PE.

Overall, there are studies supporting and others refuting the claim that prior abortions are linked to PE. While some studies endorse a protective effect and look at the paternal influence on the development of PE, other studies have found prior abortions to be an independent risk factor for PE. Furthermore, while some studies controlled for confounding factors, other studies did not, which caused pertinent limitations that may have impacted the results of the studies aforementioned. Further research focusing on the immunological implications of spontaneous abortions and how they contribute to the development of PE can lead to earlier intervention and improvement of maternal and fetal health outcomes.

Prior abortions as a risk factor for preeclampsia

While the most current research has supported the association between prior abortions and the development of PE as a protective factor for patients, there is research that refutes that claim and states that prior abortions are indeed a risk factor for PE. When analyzing these contradictory studies, it is imperative to understand the objective, methods, results, and limitations of the studies that support or negate the claim that prior abortions are protective against PE.

For instance, a cross-sectional study conducted by Sepidarkish et al. investigated 5,170 pregnant women across 103 hospitals in Tehran, Iran, and found that 252 (4.9%) of the research population experienced PE during pregnancy [17]. The analysis concluded that previous spontaneous abortions were found to be associated with PE along with a higher odds ratio of 1.28 (95% CI: 1.03-1.59, p =0.025) [17]. Through these statistical analyses, Sepidarkish et al. concluded a history of spontaneous abortion was a risk factor for the development of PE due to the increased odds ratio calculated from the study [17].

Another prospective cohort study conducted by Yemane et al. in Ethiopia followed 240 pregnant women from the first prenatal visit to delivery to investigate the potential progression of gestational hypertension (GH) to PE [18]. The study claimed that women who developed PE were likely to also have a history of prior abortion, a previous history of GH, or abnormal hemoglobin [18]. It was determined by the researchers that the study had limitations, which included the small sample size, as well as, hypercholesterolemia, antiphospholipid syndrome, thrombophilia, and multiple other blood disorders that were not assessed during this time. However, Yemane et al. stated the women enrolled in the study who had prior abortions were 3.85 times more likely to develop PE in consecutive pregnancies than their counterparts (AOR = 3.84, 95% CI: 1.07-25.0) [18].

A Chinese retrospective study consisting of 492 singleton pregnant women analyzed the relationship between first-trimester recurrent spontaneous abortions and the increased risk of complications during the antenatal period [19]. These complications included late-onset PE, oligohydramnios, early-onset fetal growth restriction, placenta percreta, increta, and accreta. Inclusion criteria for the study consisted of women with two or more abortions in early pregnancy, no karyotype abnormalities, no history of TORCH (toxoplasmosis, other agents, rubella, cytomegalovirus, and herpes simplex virus) infections, and normal insulin and thyroid levels. The data were collected using a hospital database with obstetric and perinatal information from three obstetricians. Reproductive history such as gravidity, gestational age at delivery, BMI, history of spontaneous abortions, and maternal, fetal, and placental complications were collected. The study found that first-trimester spontaneous abortions were an independent risk factor for oligohydramnios (OR: 4.62; 95% CI: 1.84-11.64), PE (OR: 3.69, 95% CI: 1.87-7,30), and cesarean section (OR: 1.7; 95% CI: 1.06-2.73) [19]. Yang et al. concluded that women with a history of spontaneous abortions should be regarded as “high risk” in future pregnancies for the occurrence of placental disorders such as PE [19].

Another retrospective cohort study executed in Scotland identified 1,561 women with initial miscarriages. Of those, 1404 suffered miscarriages in the first trimester, and 157 women experienced miscarriages in the second trimester [20]. The first unexposed cohort (group A) comprised 10,549 women who had previously delivered after 24 completed weeks in their first pregnancy and were pregnant for the second time. The second unexposed cohort (group B) included 21,118 women who delivered after 24 weeks in their first pregnancy during the study period. Further results found that 10-15% of miscarriages and spontaneous pregnancy loss were before 24 weeks. In addition, women with initial miscarriage were 3.3 times (99% CI: 2.6-4.6) more than likely to suffer from PE [20]. These data suggest that an association between prior abortions and the development of PE may exist within this population. Bhattacharya et al. stated that generally, women with a history of miscarriage have more complications in subsequent pregnancies compared to women with previous successful pregnancies; however, women with a history of miscarriage clinically had the same outcomes as primiparous women [20]. The researchers further determined that prior miscarriages are associated with an increased risk of obstetric complications, including PE, but emphasized that further research needs to be conducted to attest for confounding variables and the pathological differences between induced and spontaneous abortions and how this affects the association.

Other risk factors that affect the development of preeclampsia

Since PE is a multifactorial disease with numerous potential etiologies, it is imperative to assess other risk factors that may further implicate the suspected association between prior abortions and PE. For instance, pathologies such as CKD, pregestational diabetes, hypertension, and a decrease in hemoglobin may predispose patients to developing PE. As women may not routinely seek medical care prior to pregnancy, it is difficult to know whether these conditions may have been present for some time prior to the diagnosis of PE; therefore, it is critical to understand these other risk factors.

A study conducted by Kattah in the US demonstrated compelling evidence linking CKD as a risk factor for PE [21]. This study emphasized how CKD increases the risk of PE by multiple pathways, including the pathological mechanism of endothelial dysfunction within the vasculature, comorbid conditions such as hypertension and diabetes increasing the risk of CKD, and the disease further inducing immune and metabolic factors that may affect the reproductive system during pregnancy [21]. Moreover, a Taiwanese study found that hypertensive pregnancy disorders, in general and including PE, were associated with an increased risk of both CKD (hazard ratio (HR): 9.38, 95% CI: 7.09-12.4) and end-stage renal disease (ESRD) (HR: 12.4, 95% CI: 8.54-18.0), and further concluded that women with PE were at an even higher risk than those with GH [21]. A meta-analysis published in 2015 by Zhang et al. further evaluated the maternal complications of pregnancy in women with CKD and found that women with CKD had increased odds of developing PE (OR: 10.36, 95% CI: 6.28-17.09) [22].

GH is another theorized risk factor for PE. According to Yemane et al., GH shows a high prediction of predisposition to PE by increasing the risk of development by 17% [18]. Women who previously had a history of GH were 26 times more likely to develop PE in consecutive pregnancies than women who had not had a previous history of GH (AOR = 26.76, 95% CI: 3.03-49.08) [18].

Lastly, a decrease in hemoglobin is also a risk factor for PE. Women with hemoglobin levels between 7 and 10 g/dl are 13 times more likely to develop PE than women whose hemoglobin levels are 13 g/dl and above [18]. A study conducted in Iran showed similar findings to the association of hemoglobin and PE [17]. In women who have hypertensive disorders of pregnancy, particularly those with PE, blood volume does not increase, which results in a relatively higher hemoglobin concentration. The risk for developing pregnancy-induced hypertension was 2.46 times higher than those with lower hemoglobin concentration. However, a research analysis performed by Aghamohammadi et al. stated that high maternal hemoglobin (Hb ≥ 13.2g/dl) in the first trimester is also a risk factor for PE (OR = 2.46, 95% CI: 1-6.1) [23]. Overall, there are many conditions and health risk behaviors that need to be investigated as potential confounding variables when discussing the relationship between prior abortions and PE, as seen in Table 4.

**Table 4:** Clinical conditions and associated risk factors for preeclampsia. CI: confidence interval; P: probability; HR: hazard ratio; OR: odds ratio. References [21-23].

Condition	Quantities	CI	OR
Hypertension	≥140/90 mmHg	95% CI: 6.28-17.09	26.76
Chronic kidney disease		HR: 9.38, 95% CI: 7.09-12.4	10.36
Hemoglobin	7 and 10 g/dl	95% CI: 1-6.1	2.46
Miscarriages	>1	95% CI: 2.6-4.6	3.3
Prior abortions	>1	95% CI: 1.03-1.59, p = 0.025	3.85

Reproductive immunology

In a healthy individual, the innate and adaptive immune systems are designed to protect the body from disequilibrium. An unbalance between these immune systems can be caused by pathogens such as bacteria or foreign objects. However, one instance in which this protective shield is manipulated to tolerate non-self antigens includes the condition of pregnancy. A fetus is considered semi-allogeneic with maternal and paternal genes; the female body is designed to tolerate the fetus and help maintain the pregnancy. The uterus undergoes multiple changes to create a placenta-welcoming environment.

One of these mechanisms includes decidualization at the maternal-placental interface. The endometrial layer of the uterus differentiates into the decidua; then the fetal trophoblastic cells invade through the decidua into the myometrium. A subtype of the trophoblastic cells, fetal extravillous cytotrophoblastic cells (EVT), contribute to the remodeling of the spiral arteries by infiltrating the endothelium and muscle layers of these vessels. Once the muscle layer of these vessels is obliterated, the maternal vessels become wider with less resistance, which increases flow around the placenta [24]. EVT cells also express HLA-G ligands, which will interact with Fas-L and induce CD8+ T cell apoptosis. This interaction also inhibits cytotoxic T lymphocyte and peripheral natural killer (NK) cell actions from attacking the fetal cells [24]. The fetal trophoblastic cells have other significant roles. This direct contact between fetal and maternal tissue contributes to immunological tolerance, whereas complications with this interface are one of the possible causes of pregnancy complications such as PE and spontaneous abortion.

Another mechanism of fetal tolerance includes upregulating and downregulating immunological cells. Specifically, maternal NK cells carry an important immunological role in the detection and destruction of virally infected cells. However, they have been shown to have multiple roles in pregnancy depending on their composition. NK cells are subdivided into two groups depending on the density of CD56 surface markers. The NK cells located in the serum have a much lower proportion of CD56 than the NK cells in the uterus and are more cytotoxic. The NK cells of the uterus (uNK) have a higher density of CD56 and are more efficient at secreting cytokines. This exhibits that the morphology of the uNK cells is different from the NK cells found peripherally. These uNK cells are induced by stromal factors during pre-implantation and are upregulated during the late secretory phase of the menstrual cycle. If fertilization occurs and a blastocyst ensues, these uNK cells then secrete angiogenic growth factors, cytokines, and chemokines. These factors create a gradient that helps the trophoblast cells localize and remodel the spiral arteries [24]. uNK cells contribute further to the state of pregnancy due to their specific receptors. Specifically, uNK have activator and inhibitor receptors that belong to three main groups: lectin-C, killer immunoglobulin-like receptor (KIR), and immunoglobulin-like transcripts (ILT). The maternal KIR receptors interact with the fetal trophoblastic cell maternal and paternal HLA-C ligands. This interaction helps inhibit cytotoxic activity while promoting cytokine secretion, growth factor section, endometrium invasion, and vascular remodeling [24]. This interaction is one key to understanding the immunological tolerance between a mother and the semi-allogeneic fetus. Furthermore, this interaction is indicated to have a role in increasing the risk of PE. The inhibition of the uNK cells predisposes to PE due to their significant role in the decidual reaction, placentation, and remodeling of the spiral arteries. While the uNK cells are protective for the fetus and pregnancy, peripheral NK cells are destructive to pregnancy. However, both uNK cells and peripheral NK cells are regulated by the crown of fetal trophoblastic cells. While these fetal cells invade the myometrium of the uterus, they have human leukocyte antigens (HLA) HLA-C, HLA-E, and HLA-G. These antigens further protect the decidual reaction from being attacked by the NK cells and dendritic cells, contributing to the immunological tolerance created during pregnancy [25].

Implantation is another crucial step in the viability of pregnancy. One population of immune cells that are closely related to the female and fetal relationship includes regulatory CD4, CD8, and CD25 T cells (Tregs) [26]. In a study comparing the CD4+CD25+ Treg cell levels in 10 non-pregnant women, 19 pregnant women, and nine women with spontaneous abortions, the percentage of these cells was comparable between the non-pregnant and spontaneous abortion women, with the pregnant women’s percentage being statistically greater. Their results implied the possible contribution these cells have on the maintenance of pregnancy [26]. However, the function of CD4+ and CD25+ TReg cells is dependent on their interactions with other cells. For instance, mesodermal stromal cells (MSC) are multipotent and produce soluble factors such as transforming growth factor beta 1 (TGF𝛽1) and prostaglandin E2 (PGE2), which further contribute to the production of CD4+CD25+FOXP3+ Tregs [24]. Additionally, implantation is regulated through the complement system. The complement system is part of the innate humoral system and contributes to the clearance of pathogens, apoptotic cells, and immune complexes by the use of the membrane attack complex (MAC). This complex allows increased permeability, which eventually leads to cell lysis. The complement system is regulated via fetal cells, including the syncytiotrophoblasts, cytotrophoblasts, and EVT cells. They express proteins, decay-accelerating factor (DAF), membrane cofactor protein (MCP), and CD59. These proteins inhibit the formation of MAC and therefore inhibit the lysis of fetal cells [24], further fortifying the implantation process.

Generally, the immune system has a web of interactions between its many abilities of protection and destruction. Changes within the immunological tolerance of the fetus have been implied to contribute to pregnancy complications such as PE and spontaneous abortion. Abnormal interaction between the KIR of uNK cells and the HLA-C of the fetal EVT cells has been correlated to increased risk of PE; the interaction elicits a poorer expression and secretion of angiogenic factors placenta growth factor (PIGF), vascular endothelial growth factor (VEGF), and transforming growth factor-beta (TGF-β) from the uNK cells. In PE, there is also an increase in antiangiogenic factors from the uNK cells, such as soluble endoglin (sENG) and soluble fms-like tyrosine kinase-1 factor (sFLT1). sENG will also inhibit TGF-β from binding to its receptors thereby decreasing the nitric oxide-mediated signaling within the endothelium. Simultaneously, sFLT1 binds to VEGF and PIGF, blocking their actions. This is one proposed mechanism in which the failure of immunological tolerance can lead to PE [24]. The microscopic mother-fetus interaction strongly relies on the immunological system, whose importance is further demonstrated by paternal genetic involvement.

Immune tolerance via paternal antigen on the fetomaternal interface and its impact on subsequent pregnancies

Researchers agree that maternal immune tolerance plays a crucial role in determining a successful pregnancy. The maternal immune system tolerates the presence of a fetus expressing paternal antigens by withholding inappropriate responses against the fetus through various biochemical reactions and a system of feedback mechanisms. In current research, studies are beginning to show an association between paternal antigen-specific cells and the likelihood of miscarriages and other pregnancy complications.

With the advancements in reproductive immunological research, there have been several mechanisms found to act in combination and establish immune tolerance before the time of embryo implantation. One of the mechanisms consists of the regulation of T cells that are reactive to conceptus transplantation antigens derived from the father. In addition, another mechanism has been found associated with cytotoxic CD8+T cells causing fetal loss in mice with proinflammatory conditions, suggesting that the balance of phenotypes in paternal antigen-reactive CD8+ T cells is critical. The inability to limit T cell immunity has been associated with unexplained causes of infertility and pregnancy losses [27]. CD8+ T cells' main population, represented by the effector memory phenotype (CD8+ EM), are cells capable of potentially inducing fetal rejection. These cells were found to be the predominant cell type among the decidua immune cells, and they play a key role in feto-maternal tolerance. The researchers further analyzed the T-cell receptors B repertoire and found the expression of programmed cell death protein 1 (PD-1) in the CD8+ T cells in relation to pregnancy [27]. It was found that the most abundant population of clonally expanding CD8+ EM cells was in the decidua rather than in the periphery, which is mainly composed of naïve CD8+ T cells. It was observed that the effector memory phenotype of CD8 cells and co-inhibitory molecules like PD-1/programmed death-ligand 1 (PD-L1) were upregulated in the decidua suppressing cytotoxicity against fetal antigens [28].

The findings of Powell et al.'s study showed that CD8+ T cells recognize the fetal antigens' existence, but they are functionally suppressed by the PD-1 expression at the feto-maternal interference, allowing for suppression of fetus attack [29]. Differences were notable between the expanded CD8+ EM cells during normal pregnancy, miscarriages, and PE in terms of clonality and PD-1 expression. CD8+ effector memory phenotype tends to express PD-1 during the normal course of a pregnancy, and it was noted that the last expression of this inhibitory molecule was notably greater in those with PE or miscarriages. However, alterations in the proportion of CD8+ EM cells were not found to be responsible for pregnancy failure [28].

Furthermore, during the 1st trimester, miscarriages were found to have an increased number of clonally expanded CD8+ T EM cells with low PD-1 expression. However, during the 3rd trimester, the proportion of PD-1 expression compared to CD8+ EM cells was significantly increased in PE cases. This suggests that the antigen-specific CD8+ EM cells are less exhausted in PE and that the lack of paternal antigen-specific tolerance may be responsible [28].

Changes in paternity and its effects on preeclampsia

When discussing immune tolerance during pregnancy, it is critical to discuss the importance of semen and seminal plasma in addition to the paternal antigen. Semen is non-sterile and consists of sperm cells suspended in seminal plasma, which has a superfluous amount of immune cells. Semen also has its own microbes, which have been theorized as a potential cause of PE. Another component of seminal plasma is TGF-β, which is known to disrupt the immune response. Research completed by Kenny & Kell hypothesizes that the partner-specific protective effect of insemination is due to enhanced immune tolerance toward the partner's antigens that are found in the ejaculate as well as in the embryo [30]. For instance, when a fertile woman has repeated exposure to a male partner’s semen, the immune system increases tolerance to the microbes within that specific male’s semen [30]. The constant donation of seminal plasma allows TGF-β and prostaglandin E to induce T-regulatory cells thereby allowing the woman's immune system to acclimate to the proteins of a specific male partner. In addition to the seminal microbes causing maternal immune tolerance, these microbes may also cause endometrial inflammation, which could cause abnormal implantation in a subsequent pregnancy [30].

The association between nulliparity and PE has been widely researched. Kenny & Kell take it a step further and state that primipaternity “resets the clock” and the risk with every new partner is equivalent to a first pregnancy [30]. This is an important factor to consider because a woman can have subsequent pregnancies with the same risk for PE if each pregnancy has a different paternity. Even more so, a study in Norway analyzed data from over 1.7 million pregnancies and concluded that a male who has already fathered a preeclamptic pregnancy induces a 1.8-point increase in risk of a preeclamptic pregnancy in other primiparous mothers [31]. Overall, research has shown that maternal immune tolerance and uterine environment in addition to paternal genes and seminal microbes contribute to the likelihood of a preeclamptic pregnancy; however, additional research is needed to fully understand the impact of the semen microbe biome and the risk of pregnancy complications and increase in susceptibility to PE.

Future perspectives

Aside from previous abortions being a protective factor for PE, there are a multitude of other factors that may also induce or protect women from pregnancy complications that need future research and evaluation. For instance, for induced abortions, it remains unclear if either surgical or medical methods or if the timing of the induced abortion plays a role in future pregnancy complications [13]. In addition to this, a recent study by Mohamedain et al. concluded that spontaneous abortions have been associated with placental dysfunction and early placental failure, which are both factors in spontaneous abortion and the development of PE [11]. Prior miscarriages continue to be associated with poor maternal and perinatal outcomes; however, with a positive abortion history, there is data supporting maternal tolerance due to exposure to the fetal cells, thus decreasing the risk of developing PE. Additionally, it is important to note with studies regarding spontaneous abortions, the women may have a bias when recalling the number of previous miscarriages.

Generally speaking, it is important for future studies to assess and evaluate the differences in outcomes between induced and spontaneous abortions, and their association with PE and other pregnancy pathologies as it would be useful in establishing a thorough understanding of these relationships. Furthermore, within the obstetric and gynecological research community, it has been widely accepted that nulliparous women are more commonly associated with a higher risk for PE and other pregnancy complications. However, future studies that can evaluate and assess both nulliparous and multiparous women in regards to the likelihood of developing PE and similar pathologies with consideration of placental dysfunction, maternal immune tolerance, paternal antigens, and the seminal microbe biome are imperative to understanding the broader picture of the development of conditions such as PE in the setting of a positive abortion history.

## Conclusions

While current literature readily supports a positive abortion history as a protective factor for the pregnancy complication of PE, it is important to acknowledge the research that supports the contrary position that previous abortions are a risk factor for PE. In addition, understanding confounding variables in these studies and other risk factors that may predispose women to developing pregnancy complications such as PE is crucial to further understanding the intricacies of these types of complications and formulating adequate treatment plans for these at-risk patients.

Within the last several decades, the field of reproductive immunology research has continued to yield new information regarding maternal immune tolerance and its relationship with the development of pregnancy complications. Understanding the pathological and immunological mechanisms that occur at the fetomaternal interface during pregnancy, as well as the role of the paternal antigen and seminal microbes, has been deemed imperative to widening the knowledge of pregnancy pathological conditions. Overall, additional studies will be needed to further understand the association between prior abortions as a protective factor for PE and the reproductive immunology that supports this relationship.
